# Outcomes of Endoscopic Treatment for Post-Metabolic and Bariatric Surgery Leaks: Beyond The ‘Binary’ Notion to a Need for Contextual Reporting of Success Rates

**DOI:** 10.1007/s11695-025-08083-1

**Published:** 2025-07-15

**Authors:** Walid El Ansari, Mohamed Hany

**Affiliations:** 1 College of Medicine, Ajman University, Ajman, United Arab Emirates; 2 Department of Surgery, Medical Research Institute, Alexandria University, Alexandria, Egypt; 3 Madina Women’s Hospital, Alexandria University, Alexandria, Egypt

Dear Editor,

We read with deep interest and were greatly intrigued by Minakari et al.’s work entitled “Success Rate of Endoscopic Treatment for Post‐Sleeve Gastrectomy Leaks: A Systematic Review and Meta‐Analysis” [1]. We applaud the authors on this timely contribution to the metabolic and bariatric surgery (MBS) literature. Their meta-analysis provides important insights on the efficacy and safety of endoscopic interventions for the management of post-sleeve leaks, reporting an overall success rate of 88%. We note that Minakari and colleagues appropriately reported several important secondary outcomes, including the mean number of endoscopies and stents per patient, and complication rates. These contributions provide valuable baseline information for the field.

However, we believe that connecting these secondary outcomes more explicitly to reported success rates would enhance the utility and interpretability of a given success rate. Below, we highlight a few points for consideration for future research of success rates (SRs) of endoscopic treatments for post-MBS leakage.

## Introduction

Minakari et al.’s [1] study focused on the assessment of SRs of endoscopic treatment. It also provided a definition of successful outcome or successful management, as the number of patients completely healed through endoscopic interventions without resorting to surgery. Whilst this is a clear and important outcome, it appears not to consider the sequence of events that happen until success, as defined, is achieved. Several factors might be worth taking into account.

## Methodological Concerns

*Temporal considerations (timeframe, follow-up)*: For instance, the time horizon during which success was achieved in different studies that were included would appear to be important.

*Procedural complexity (number of interventions, stent usage)*: Likewise, the number of endoscopic manoeuvres undertaken per patient in different studies until success was achieved would be of value. Similarly, the number of stents used per patient until surgery was declared as not required would be beneficial to know. Was resolution of the leak achieved after one stent, two stents, or more?

*Treatment sequence (first line vs. adjunctive)*: An additional aspect is whether stent placement/endoscopic treatment was the first management for the leak or otherwise, as previous treatments will likely influence the outcome of the index stent placement.

*Data handling (lost to follow-up, incomplete data)*: Furthermore, information on how patients who were lost to follow-up or expired during the study period after receiving endoscopic treatment, or those with incomplete data, were handled in the calculations of success or failure rates is also noteworthy.

Taken together, these aspects suggest a need for contextual reporting of the outcome rates of endoscopic treatment after MBS leakage.

### Contextual Reporting

SRs are important to know. But it is more critically important to consider the ‘landscape’ surrounding a computed SR. A ‘naked’ SR provided alone in isolation, with no information on the prevailing context becomes one that is difficult to interpret, and even more arduous to compare with another SR reported from elsewhere or reported by another review a few years from now. Such a ‘sequestered’ SR becomes akin to a skeleton without flesh, or a post-bariatric complication rate without, e.g., a reference timeframe.

### Why Contextual Reporting?

The utility of a given SR is greatly reduced when it is not accompanied by important complementing data on e.g., the time limit during which it was measured, an indication of the number of endoscopic manoeuvres that were undertaken until success ensued, understandings of the number of stents that were used to stop leakage, and/or data about whether it was the first line of treatment or was otherwise complementing other prior management strategies.

Longer time horizons during which success is measured could inflate SRs. The impact of varying timeframes on success rate reporting has been demonstrated in endoscopic intervention research for leakage. For instance, a study of stents for post-MBS staple line leaks noted that the definition of success in their study was stricter than in some other studies, as four patients who had leak resolutions more than one week after stent removal, but without additional interventions, were not considered successful in their study [2]. If these patients had been defined as successful, as in several other studies, their reported SR would have been higher [2]. This highlights how temporal aspects of definitions can significantly affect the reported outcome rates.

Similarly, a sequence of endoscopic manoeuvres might increase the chances of success up to a limit, and the use of several successive stents could increase the likelihood of sealing the leakage. Indeed, Minakari and colleagues [1] rightly acknowledge that they were unable to analyze stent migration based on the number of stents placed, due to limited data. Others, in a study of stents in the treatment of leaks complicating laparoscopic sleeve gastrectomy, reported that clinical success achieved increased from 78.13% to 89.06% and 93.75% for the first round, second round, and third round of stenting, respectively [3].

### Necessary But Not Sufficient

Such contextual variables could bias a measured success estimate, driving a rate up or down. Hence, a stand-alone SR of endoscopic treatment is necessary but not sufficient. It is a useful, albeit a ‘crude’ indicator which limits its practical usefulness and value. Conversely, adding the abovementioned information to a given SR greatly enhances its utility, comparability, and meaningfulness. In their secondary outcomes, Minakari and colleagues [1] provided information on the number of endoscopic interventions required per patient. Whilst this is useful, however, failing to connect it up to the SR greatly reduces its usefulness.

### ‘Binary’ Notion of Outcomes of Endoscopic Treatment of Post-MBS Leaks

Bundling the outcomes of endoscopic treatment for post‐bariatric leaks into a bipolar situation of no requirement for surgery (i.e., success) vs. requirement for surgery (i.e., failure) appears to create an over reductionist scenario. By its very nature, such a binary scenario implicitly assumes that all cases of success are inherently equal, regardless of the time required, the number of maneuvers, the number of stents, and the presence or absence of any other management undertaken prior to the index endoscopic treatment. This situation does not represent real life, as it adopts a ‘broad brush’ approach to computing success and falls short of providing the fine granularity of detail surrounding a given SR.

With no important elements attached to a SR to characterize it, present and future comparisons of the rate with another are likely to be akin to comparing apples with oranges…meaningless. Without ‘anchoring’ a SR to such variables or other defining factors in order to change it from a unidimensional to a bidimensional estimate, comparisons of rates will be imprecise, inaccurate, and possibly misleading. Extrapolating the effects of such imprecision further, future models to predict success or failure of endoscopic treatment for leakage after bariatric surgery will probably yield inconsistent findings. This is partly because the predicted outcome (success) is, in its current state, not a single homogenous well-defined entity, but rather, heterogenous and loosely bunched sets of entities. Such undertakings are likely to add more inconsistencies to the evidence base rather than progress it forward with robust certainty.

### Moving Beyond Binary Outcomes: Future Directions

What success rates truly require is a unit! Characterizing success by a candidate unit, e.g., by a time horizon, or per endoscopic maneuver conducted, or per stent used could be the first step to a more precise calculation of SRs. Such undertaking would be the initial building block of all analyses that come thereafter, including comparisons, logistic regression predictions, clinical scores to triage patients, or machine learning models.

## Conclusion

Minakari et al. have presented an illuminating review on the success rate of endoscopic treatment for post‐sleeve leaks. We commend their contribution on this important topic for bariatric practitioners and patients, and propose our comments and suggestions in a constructive atmosphere of academic discourse. Attention to these issues could extend and enhance the current knowledge and our comprehension of the success of endoscopic treatment for post‐bariatric leaks, ultimately resulting in higher quality patient care. We thank the authors for sparking a debate and look forward to future research building on their work.

## Data Availability

No datasets were generated or analysed during the current study.
